# Valve disease in cardiac amyloidosis: an echocardiographic score

**DOI:** 10.1007/s10554-023-02901-2

**Published:** 2023-06-21

**Authors:** Alberto Aimo, Iacopo Fabiani, Agnese Maccarana, Giuseppe Vergaro, Vladyslav Chubuchny, Emilio Maria Pasanisi, Christina Petersen, Elisa Poggianti, Alberto Giannoni, Valentina Spini, Claudia Taddei, Vincenzo Castiglione, Claudio Passino, Marianna Fontana, Michele Emdin, Lucia Venneri

**Affiliations:** 1 Cardiology Division, Fondazione Toscana Gabriele Monasterio, Pisa, Italy; 2 Interdisciplinary Center for Health Sciences, Scuola Superiore Sant’Anna, Piazza Martiri della Libertà 33, 56124 Pisa, Italy; 3 National Amyloidosis Centre, University College London, Royal Free Campus, London, UK

**Keywords:** Cardiac amyloidosis, Diagnosis, Echocardiography, Score, Valve disease

## Abstract

Cardiac amyloidosis (CA) may affect all cardiac structures, including the valves. From 423 patients undergoing a diagnostic workup for CA we selected 2 samples of 20 patients with amyloid transthyretin (ATTR-) or light-chain (AL-) CA, and age- and sex-matched controls. We chose 31 echocardiographic items related to the mitral, aortic and tricuspid valves, giving a value of 1 to each abnormal item. Patients with ATTR–CA displayed more often a shortened/hidden and restricted posterior mitral valve leaflet (PMVL), thickened mitral chordae tendineae and aortic stenosis than those with AL–CA, and less frequent PMVL calcification than matched controls. Score values were 15.8 (13.6–17.4) in ATTR–CA, 11.0 (9.3–14.9) in AL–CA, 12.8 (11.1–14.4) in ATTR–CA controls, and 11.0 (9.1–13.0) in AL–CA controls (p = 0.004 for ATTR- vs. AL–CA, 0.009 for ATTR–CA vs. their controls, and 0.461 for AL–CA vs. controls). Area under the curve values to diagnose ATTR–CA were 0.782 in patients with ATTR–CA or matched controls, and 0.773 in patients with LV hypertrophy. Patients with ATTR–CA have a prominent impairment of mitral valve structure and function, and higher score values. The valve score may help identify patients with ATTR–CA among patients with CA or unexplained hypertrophy.

**Supplementary Information:**

The online version contains supplementary material available at 10.1007/s10554-023-02901-2.

## Background

Amyloidosis is a systemic disorder characterized by extracellular deposition of insoluble fibrils. The vast majority of cases of cardiac amyloidosis (CA) are caused by the accumulation of immunoglobulin light-chain (AL–CA) or transthyretin (ATTR–CA), a carrier for thyroxine and retinol-binding protein [1]. On echocardiographic examination, some of the most evident manifestations of CA are increased left ventricular (LV) wall thickness (pseudohypertrophy), diastolic dysfunction, depressed LV systolic function with relative preservation of the apex (apical sparing) [2, 3]. Nonetheless, amyloid deposition affects all cardiac chambers, as partially acknowledged by the most established echocardiographic diagnostic score, including tricuspid annular plane systolic excursion as an item [4]. Valvular amyloidosis has come to attention because of the association between severe aortic stenosis and CA [5–8], and the detection of amyloid deposits in surgically explanted aortic valve [9]. Scattered evidence on valve disease derives from echocardiographic studies. Patients with CA have been reported to have thickened mitral or aortic valves in up to 31% of cases [10–12]; mitral regurgitation is often mild to moderate [13], but hemodynamically significant mitral or tricuspid regurgitation can be found in 50% of patients in more advanced stages [11]. In this study we performed the first systematic assessment of the echocardiographic features of valvular CA. We then synthesized these features in a score and evaluated its diagnostic and prognostic value.

## Methods

### Patient population

We evaluated 423 consecutive patients referred to the Fondazione Toscana Gabriele Monasterio (FTGM), Pisa, Italy from 2015 to 2020 for a diagnostic work-up for suspected CA. Patients were referred because of proven systemic AL amyloidosis (n = 60, 14%) or unexplained increased LV wall thickness on echo (interventricular septal or posterior wall thickness ≥ 12 mm) (n = 363, 86%), together with clinical and/or laboratory findings compatible with CA [4]. Patients underwent a complete diagnostic work-up in agreement with the diagnostic algorithm by Gillmore et al. [14]. ATTR–CA was diagnosed when patients had grade 2–3 cardiac uptake on diphosphonate scintigraphy in the absence of monoclonal gammopathy or an endomyocardial biopsy (EMB) containing ATTR amyloid [14]. AL–CA was defined by an EMB containing AL amyloid, or the combination of characteristic features on echocardiography/cardiovascular magnetic resonance (CMR) [15] and histologically proven systemic AL amyloidosis on a non-cardiac biopsy [16]. CA was diagnosed in 261 patients (62%; ATTR–CA, n = 144; AL–CA, n = 117).

For the purposes of this study, we randomly selected 2 samples of patients with ATTR–CA or AL–CA (n = 20 each), and we matched them by age and sex with 2 samples of patients with CA excluded. Patients with prosthetic valves or an history of valvuloplasty were excluded both from the CA and control groups. The study protocol conformed to the 1975 Declaration of Helsinki and was approved by the Institutional Human Research Committee. All patients provided written informed consent.

### Echocardiography

Each echocardiogram was performed by an expert imager using a commercially available system (GE Vivid E95 Medical Systems, Horten; Philips IE33/Epiq—Philips Medical Systems, Palo Alto, California, USA) equipped with a 1.5–3.6-MHz transducer (M4S; M5S GE; X51 Philips). Chamber volumes, LV mass, LV diastolic dysfunction, valve regurgitation and stenosis, dimensions and longitudinal function of the RV were evaluated according to current recommendations [17–20]. LV hypertrophy was defined as LV mass index (LVMI) ≥ 115 g/m^2^ (men) or ≥ 95 g/m^2^ (men) [17]. Speckle-tracking echocardiography was retrospectively performed in December 2020 on stored acquisitions by expert operators (I.F., V.S.) blinded to the final diagnosis. Among the consecutive patients included in this study, those meeting the following criteria did not undergo STE assessment: mitral valvular prosthesis; extensive mitral annular calcifications; device for atrial septal occlusion; poor acoustic window limiting the deformation analysis of ≥ 2 segments in each window. Each center analyzed strain parameters using off-line semi-automatic 2D strain software, with validated inter-vendor consistency (2D Cardiac Performance Analysis, TomTec-Arena version 4.6, TomTec Imaging Systems, Unterschleissheim, Germany; EchoPAC 12.0, GE, USA). 2D grey-scale apical 4-, 2- and 3-chambers views were acquired during 3 consecutive cardiac cycles, with a frame rate > 50 frames/s. The endocardial border was manually traced on an end-systolic frame. The software automatically generated an epicardial line to create the region of interest in the 4-, 2- and 3-chamber views, which was manually corrected when needed. The myocardium was automatically divided into 16 segments. A deformation curve for each segment was generated, and a mean curve was derived from the average of the segments. Normal global longitudinal strain (GLS) values are − 21.7 ± 2.5% (lower reference limit − 16.7) in men and − 23.0 ± 2.7% (lower reference limit − 17.8) in women [21]. For speckle-tracking analysis of the other chambers, see the Supplemental Material.

### The valve score

**Fig. 1:**
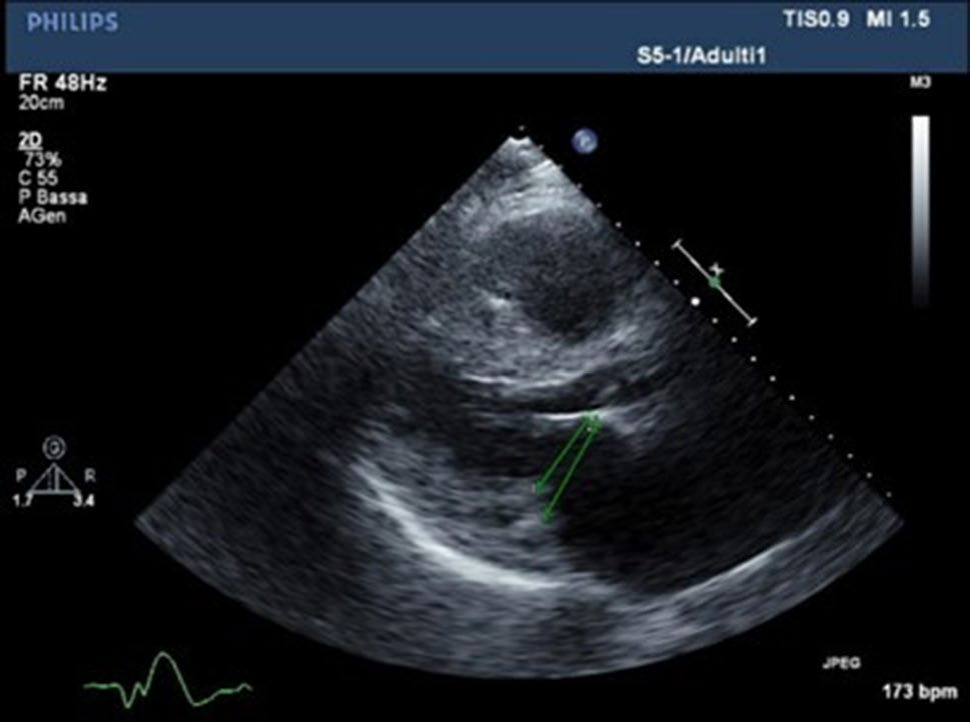
Standard and modified anteroposterior diameter of the mitral valve annulus in a in a 75-year-old man with cardiac transthyretin amyloidosis. Parasternal long-axis view. The modified diameter of the mitral valve annulus is marked as 1, while the standard diameter as 2. See text for further details

A score providing a comprehensive assessment of the mitral, aortic and tricuspid valves was devised by echocardiographers with a specific expertise in CA (L.V., I.F.), and employed in October 2021 on stored echocardiograms. The pulmonary valve was not included because it is examined in a single, parasternal short axis view in standard echocardiograms (not allowing to assess properly its structure and function), and because mild regurgitation was the only functional abnormality found in our patients (n = 51, 64%). We considered the length, thickness, restriction and calcification of the posterior mitral valve leaflet (PMVL), and the thickness, restriction and calcification of the anterior mitral valve leaflet; in our experience, the anterior mitral valve leaflet is not shortened in patients with CA, therefore its length was not included as a score item. The anteroposterior (AP) diameter of the mitral valve annulus is measured in the parasternal long-axis view at end-diastole, both as the distance between the insertion points of the leaflets (standard AP diameter) and as the distance between the insertion point of the anterior leaflet and leaflet intersection with the posterior wall (modified AP diameter, Fig. 1). Calcification of the mitral annulus was also included, as well as chordal and papillary muscle thickness. Cusp thickness, restriction and calcification, and function of the aortic valve were evaluated. As for the tricuspid valve, the septal leaflet and the anterior or posterior leaflet (i.e., the non-septal leaflet in the apical 4-chamber view) were described. The annular diameter and calcification, chordae tendineae and valve function were assessed as for the mitral valve. The items for each element, the possible values of each item, and the corresponding score points are listed in Table 1. For each item, either 2 options (0, normal; 1, abnormal) or 3 options (0, normal; 0.5, mildly abnormal; 1, overtly abnormal) are proposed. The only exception is represented by the items related to calcification (0, presence of calcification; 1, absence of calcification), based on our observation that calcification of valve structures is quite limited in patients with CA. Score values range from 0 to 31. Score values were calculated by an experienced echocardiographer (I.F.). Intra- and interobserver variability was evaluated in a random sample of 10 patients, involving a second echocardiographer (A.M.).

**Table 1:** The valve score

Valve	Item	Description	Point
Mitral valve			
Posterior leaflet	Thickness	Not thickened	0
		Thickened	1
	Length	Normal	0
		Short	0.5
		Hidden	1
	Restriction	Not restricted	0
		Only base	0.5
		Restricted	1
	Calcification	Absent	1
		Present	0
Anterior leaflet	Thickness	Not thickened	0
		Thickened	1
	Restriction	Not restricted	0
		Only base	0.5
		Restricted	1
	Calcification	Absent	1
		Present	0
Annulus	AP–APm (absolute value)	< 3	0
		≥ 3 and < 5	0.5
		≥ 5	1
	Calcification	Absent	1
		Present	0
Chordae tendineae	Thickness	Not thickened	0
		Thickened	1
Papillary muscles	Thickness	Not thickened	0
		Thickened	1
Valve function	Stenosis	Absent	0
		Mild	0
		Moderate	1
		Severe	1
	Regurgitation	Absent	0
		Mild	0
		Moderate	1
		Severe	1
Aortic valve			
Cusps	Thickness	Not thickened	0
		Thickened	1
	Restriction	Not restricted	0
		Restricted	1
	Calcification	Absent	1
		Present	0
Valve function	Stenosis	Absent	0
		Mild	0
		Moderate	1
		Severe	1
	Regurgitation	Absent	0
		Mild	0
		Moderate	1
		Severe	1
Tricuspid valve			
Septal leaflet	Thickness	Not thickened	0
		Thickened	1
	Length	Normal	0
		Short	0.5
		Hidden	1
	Restriction	Not restricted	0
		Only base	0.5
		Restricted	1
	Calcification	Absent	1
		Present	0
Antero-posterior leaflet	Thickness	Not thickened	0
		Thickened	1
	Length	Normal	0
		Short	0.5
		Hidden	1
	Restriction	Not restricted	0
		Only base	0.5
		Restricted	1
	Calcification	Absent	1
		Present	0
Annulus	Calcification	Absent	1
		Present	0
Chordae tendineae	Thickness	Not thickened	0
		Thickened	1
Papillary muscles	Thickness	Not thickened	0
		Thickened	1
Valve function	Stenosis	Absent	0
		Mild	0
		Moderate	1
		Severe	1
	Regurgitation	Absent	0
		Mild	0
		Moderate	1
		Severe	1

*AP* anteroposterior, *SAM* systolic anterior motion

### IWT and AMYLI scores

We also compared the yield of the valve score, the increased wall thickness (IWT) score and the AMYLoidosis Index (AMYLI) score to diagnose ATTR–CA. The IWT score is a tool to diagnose CA in patients with unexplained LV hypertrophy referred to a diagnostic work-up for CA. It includes the following variables: relative WT (RWT: 2 * posterior WT in end-diastole/LV end-diastolic diameter]; E/e′ ratio; tricuspid annular plane systolic excursion; GLS; systolic apical to base ratio)^4^ (Supplemental Table 2). The AMYLI score is a simplified version of this score, previously developed and validated, defined as the product of RWT and E/e′ [22].

### Laboratory evaluation

See the Supplemental Material.

### Follow-up

Patients were followed at the FTGM in a dedicated outpatient clinic. Information on all-cause death, cardiovascular death and heart failure (HF) hospitalization was collected from FTGM electronic health records or phone calls to patients or their relatives, performed in November 2021.

### Statistical analysis

Statistical analysis was performed using IBM SPSS Statistics (version 22, 2013). Normal distribution was assessed through the Shapiro–Wilk test. As all variables had a non-normal distribution, they were presented as median and interquartile interval. Mean differences among groups were evaluated through the Kruskal–Wallis one-way analysis of variance, applying the Bonferroni correction for multiple comparisons (n = 3). Discrete variables were compared by the χ^2^ test with Yates correction or the Fisher exact test. Area under the curve (AUC) values were calculated, and the best cut-off was defined through the Youden index. AUC values were compared through the De Long’s test. Predictors of ATTR–CA were searched through uni- and multivariable logistic regression analysis. Intra- and inter-observer variability was evaluated through intraclass correlation coefficients (ICC) and the corresponding 95% confidence intervals (CI). Two tailed p values < 0.05 were deemed significant (or < 0.017 after Bonferroni correction).

## Results

### Patient population

The main characteristics of the 4 patient groups are reported in Table 2. Patients with ATTR–CA had a median age of 81 years (75–84), and 90% were men, while patients with AL–CA were aged 76 years (67–82), and three quarters of them were men. Patients with ATTR–CA had greater degrees increase in LV mass than patients with AL–CA or controls matched to ATTR–CA patients based on age and sex. Compared to matched controls, they had also a more prominent concentric hypertrophy (as expressed by RWT values), a worse diastolic function (in terms of E/e′ ratio), and a more dysfunctional LA (as demonstrated by lower peak LA longitudinal strain). Patients with AL–CA were more similar to their matched controls, without significant differences except for a lower prevalence of hypertension (Table 2).

**Table 2:** Patient characteristics

	ATTR–CA n = 20	AL–CA n = 20	ATTR–CA control n = 20	AL–CA control n = 20	ATTR–CA vs. AL–CA p	ATTR–CA vs. ATTR–CA control p	AL–CA vs. AL–CA control p
Age (years)	81 (75–84)	76 (67–82)	81 (75–84)	76 (67–82)	0.043	1	1
Men, n (%)	18 (90)	15 (75)	18 (90)	15 (75)	0.212	1	1
NYHA I/II/III/IV, n (%)	5, 8, 7, 0 (25, 40, 35, 0)	6, 8, 5, 1 (30, 40, 25, 5)	8, 7, 5, 0 (40, 35, 25, 0)	1, 11, 8, 0 (5, 55, 40, 0)	0.700	0.579	0.125
Hypertension, n (%)	15 (75)	12 (60)	17 (85)	19 (95)	0.311	0.429	**0.008**
Diabetes, n (%)	3 (15)	4 (20)	3 (15)	4 (20)	0.677	1	1
NT-proBNP (ng/L)	3204 (2024–5917)	4,330 (1374–23,363)	3575 (692–5401)	2327 (1113–4474)	0.380	0.270	0.113
hs-TnT (ng/L)	51 (38–121)	55 (37–134)	44 (30–65)	33 (19–57)	0.707	0.336	0.033
eGFR (mL/min/1.73 m^2^)	62 (33–71)	67 (51–83)	58 (44–76)	57 (49–72)	0.124	0.784	0.230
Atrial fibrillation/flutter/tachycardia, n (%)	9 (45)	7 (35)	8 (40)	10 (50)	0.507	0.744	0.327
IVS (mm)	17 (14–19)	14 (12–16)	14 (12–15)	15 (12–16)	**0.004**	** < 0.001**	0.718
PW (mm)	14 (13–16)	13 (11–15)	12 (11–13)	13 (11–15)	**0.006**	** < 0.001**	0.718
LVEDVi (mL/m^2^)	51 (46–65)	55 (40–70)	55 (49–72)	65 (56–78)	0.923	0.184	0.033
LVESVi (mL/m^2^)	27 (22–30)	25 (19–34)	24 (13–35)	28 (19–38)	0.817	0.538	0.647
LVEF (%)	50 (44–57)	51 (42–60)	56 (46–68)	59 (51–68)	0.968	0.102	0.049
GLS (%)	− 13 (− 17 to − 7)	− 10 (− 15 to − 9)	− 13 (− 16 to − 6)	− 15 (− 17 to − 11)	0.531	0.815	0.038
LVMI (g/m^2^)	157 (148–188)	141 (111–157)	127 (106–154	165 (137–178)	**0.006**	**0.004**	0.091
MSR	13.6 (10.0–21.3)	13.2 (7.7–17.2)	9.8 (7.8–25.0)	10.7 (7.4–15.3)	0.552	0.271	0.454
RWT	0.6 (0.5–0.7)	0.5 (0.4–0.6)	0.5 (0.4–0.5)	0.5 (0.4–0.6)	0.081	** < 0.001**	0.301
E/e′	14 (11–18)	14 (10–21)	10 (9–16)	13 (11–15)	0.923	**0.030**	0.380
LAVI (mL/m^2^)	49 (39–56)	44 (38–48)	44 (37–51)	47 (39–51)	0.141	0.265	0.327
PALS (%)	6.9 (5.3–12.0)	7.7 (3.3–10.5)	16.2 (11.6–19.7)	11.3 (6.7–14.5)	0.631	**0.001**	0.121
RV diameter (mm)	27 (24–30)	28 (25–31)	28 (26–31)	29 (27–31)	0.565	0.355	0.165
TAPSE (mm)	17 (14–20)	17 (14–20)	19 (14–21)	19 (16–23)	0.857	0.640	0.191
RV strain (%)	− 16 (− 17 to − 11)	− 12 (− 16 to − 9)	− 17 (− 22 to − 13)	− 15 (− 18 to − 10)	0.206	0.220	0.377
Systolic PAP (mmHg)	43 (34–49)	39 (35–48)	38 (34–48)	48 (34–58)	0.749	0.647	0.488
RA diameter (mm)	50 (43–52)	49 (44–53)	46 (40–52)	45 (42–51)	0.925	0.478	0.258
RA strain (%)	0.8 (0.7–1.0)	0.8 (0.6–0.9)	0.7 (0.4–0.8)	0.6 (0.5–0.7)	0.959	0.107	0.038

The Bonferroni correction was applied to account for multiple comparisons (n = 4); significant p values (< 0.017) are highlighted in bold

*AL* amyloid light-chain, *ATTR* amyloid transthyretin, *eGFR* estimated glomerular filtration rate, *IVS* interventricular septum, *LAVI* left atrial volume index, *LVEF* left ventricular ejection fraction, *LVEDVi* left ventricular end-diastolic volume index, *LVESVi* left ventricular end-systolic volume index, *LVMI* left ventricular mass index, *MSR* mass-to-strain ratio, *NT-proBNP* N-terminal pro-B-type natriuretic peptide, *NYHA* New York Heart Association, *PAP* pulmonary artery pressure, *PW* posterior wall, *RA* right atrial, *RV* right ventricular, *RWT* relative wall thickness, *TAPSE* tricuspid annular plane systolic excursion

### The valve score

**Fig. 2:**
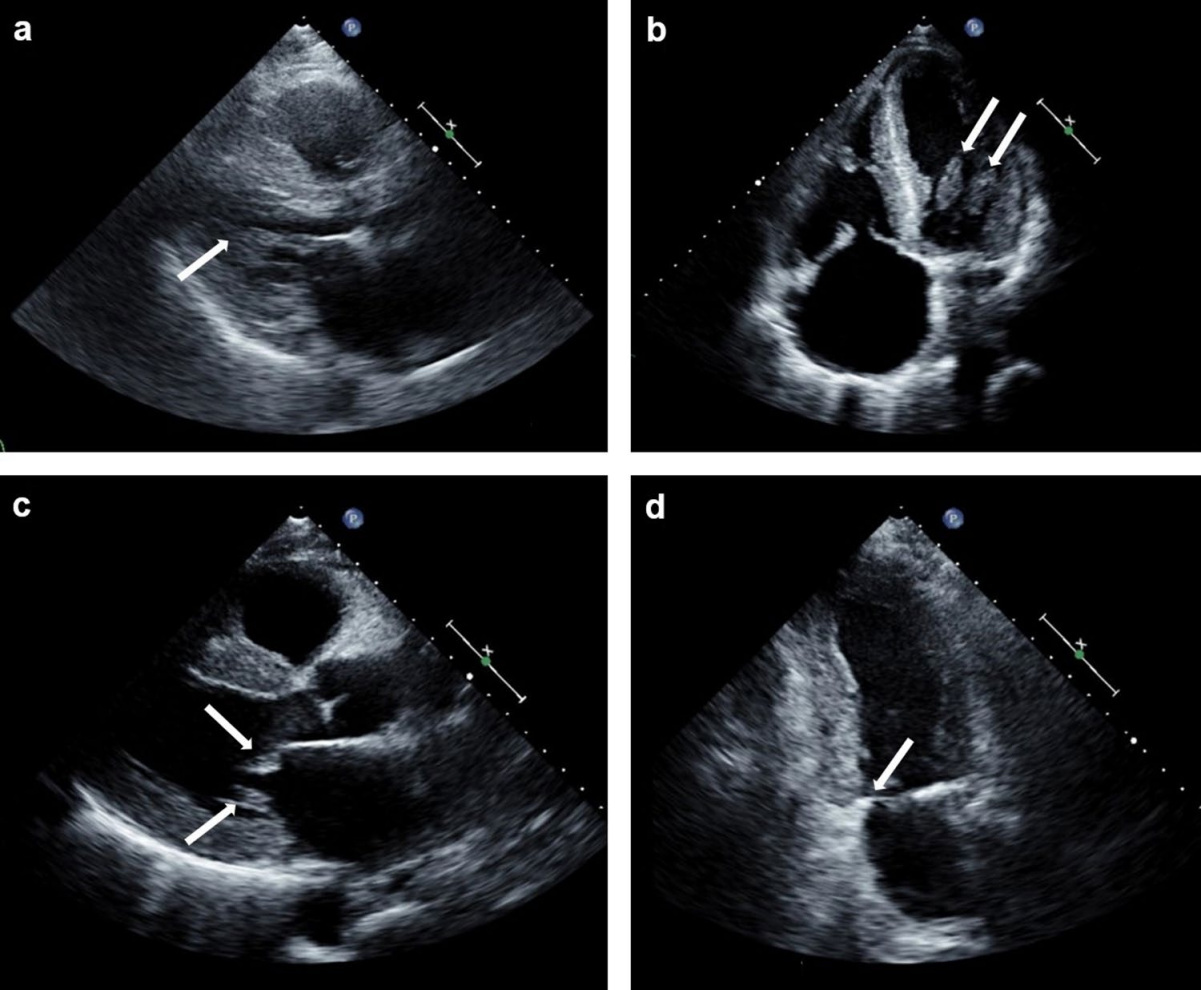
Features of mitral valve involvement in three men with cardiac transthyretin amyloidosis.** a** and **b** Thickened papillary muscles on a parasternal long-axis view (**a**) and an apical 4-chamber view (**b**), **c** parasternal long-axis view showing mitral leaflets thickening, and **d** apical 4 chambers view showing an almost hidden posterior mitral leaflet

**Fig. 3:**
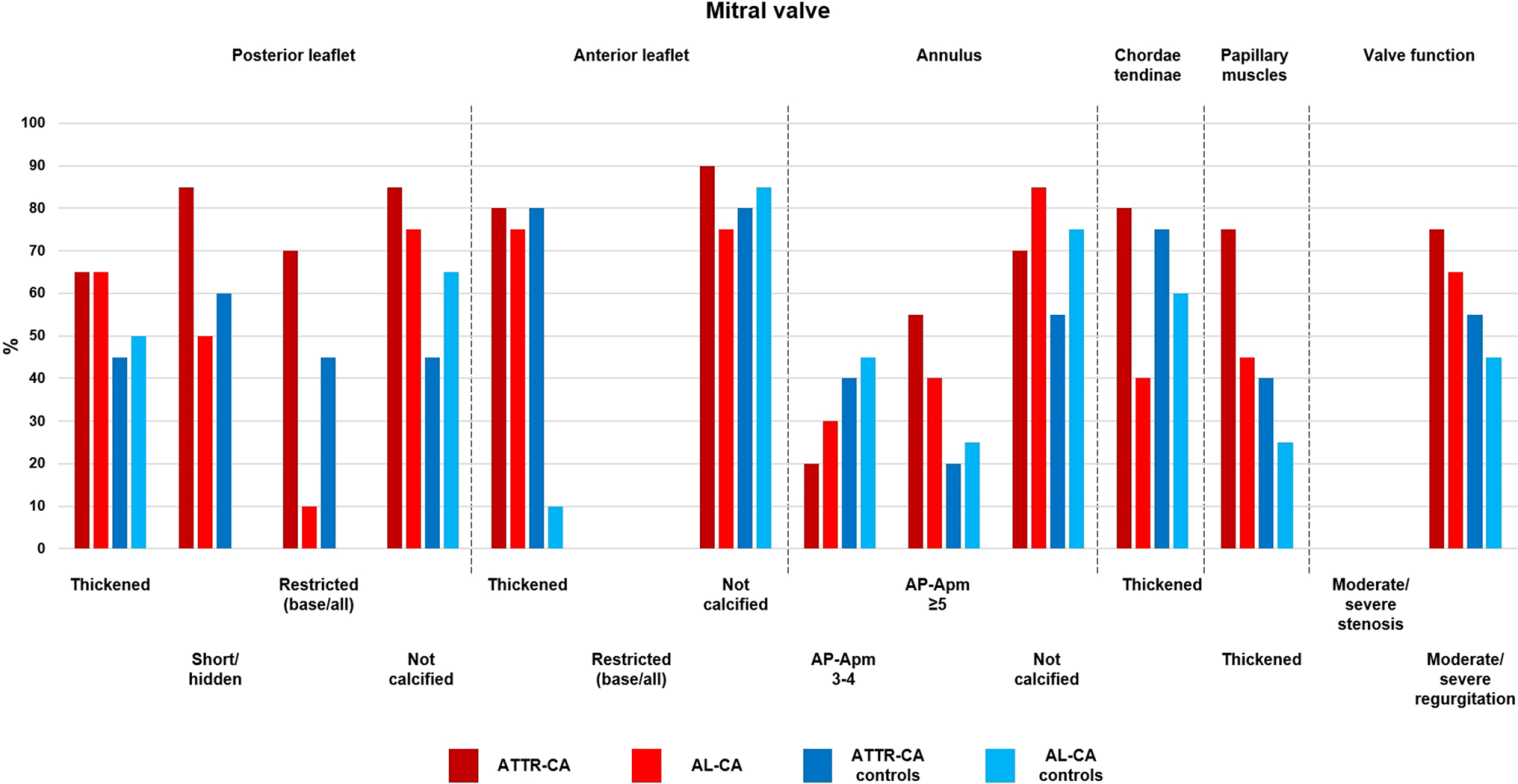
Valve score items: mitral valve. *AL* amyloid light-chain, *AP* antero-posterior, *ATTR* amyloid transthyretin, *CA* cardiac amyloidosis

**Fig. 4:**
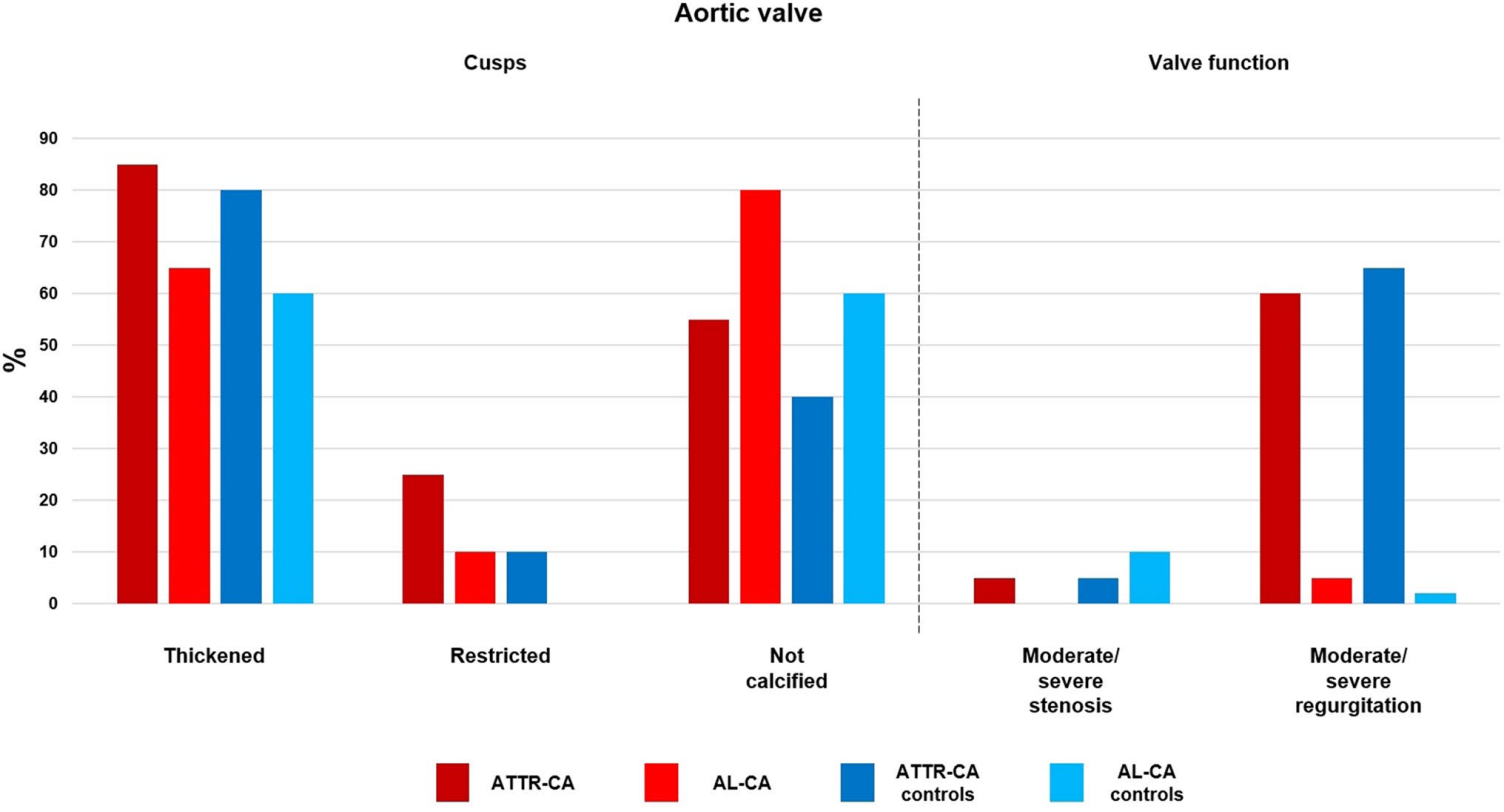
Valve score items: aortic valve. *AL* amyloid light-chain, *AP* antero-posterior, *ATTR* amyloid transthyretin, *CA* cardiac amyloidosis

**Fig. 5:**
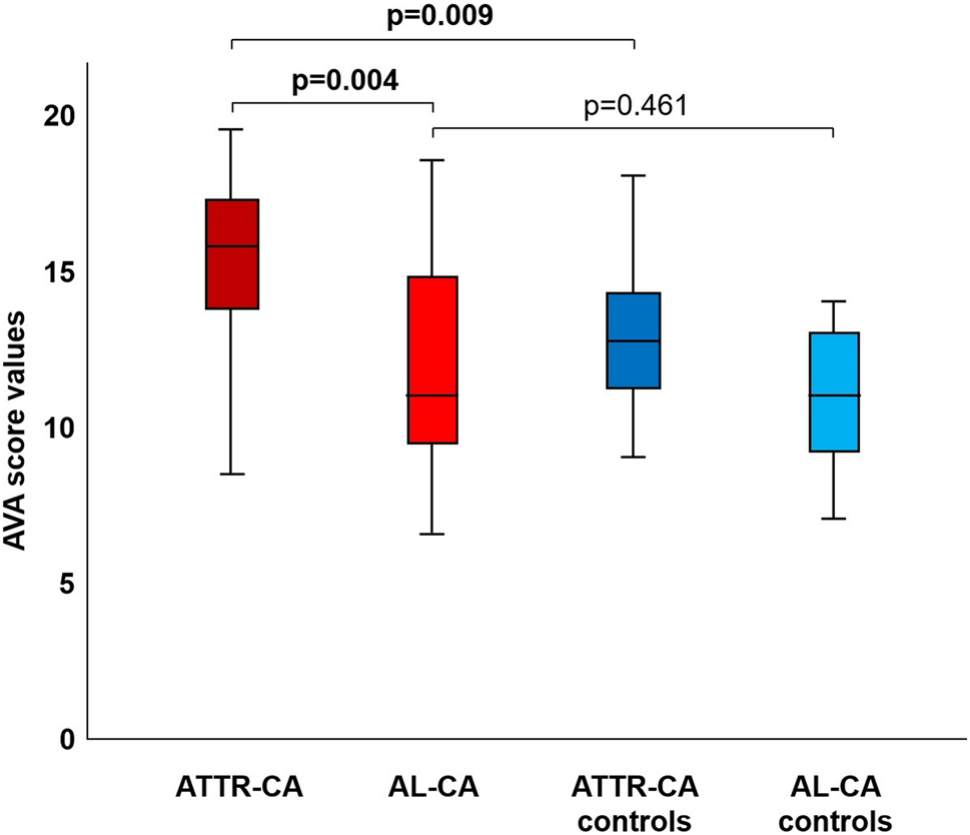
Amyloid valve score values in patients with amyloid transthyretin (ATTR) or light-chain (AL) cardiac amyloidosis (CA) and matched controls. The 14 cut-off value was selected based on the Youden index; see text for details

We then calculated the valve score values in the four groups. When considering single parameters, patients with ATTR–CA more often showed a shortened or hidden and restricted PMVL than patients with AL–CA, as well as thickened mitral chordae tendineae and a stenotic aortic valve. The only significant difference between ATTR–CA and their matched controls was represented by less frequent calcification of the PMVL. Furthermore, patients with AL–CA displayed more often a short or hidden PMVL than their matched controls (Table 3; Figs. 2, 3, 4). Additionally, patients with ATTR–CA had higher mitral score values than those with AL–CA or than ATTR–CA controls (Table 3). Global score values were 15.8 (13.6–17.4) in patients with ATTR–CA, 11.0 (9.3–14.9) in those with AL–CA, 12.8 (11.1–14.4) in ATTR–CA controls, and 11.0 (9.1–13.0) in AL–CA controls (p values: 0.004 for ATTR–CA vs. AL–CA, 0.009 for ATTR–CA vs. matched controls, and 0.461 for AL–CA vs. matched controls) (Table 3; Fig. 5). A very low intra- and interobserver variability in score calculation was observed (ICC intra: 0.97, 95% CI 0.89–0.97; ICC inter: 0.93, 95% CI 0.87–0.94).

**Table 3:** Valve score items and values

Valve	Item	Description	ATTR–CA n = 20	AL–CA n = 20	ATTR–CA controls n = 20	AL–CA control n = 20	ATTR–CA vs. AL–CA p	ATTR–CA vs. ATTR–CA control p	AL–CA vs. AL–CA control p
Mitral valve									
Posterior leaflet	Thickness	Not thickened, n (%)	7 (35)	7 (35)	11 (55)	10 (50)	1	0.204	0.337
		Thickened, n (%)	13 (65)	13 (65)	9 (45)	10 (50)			
	Length	Normal length, n (%)	3 (15)	10 (50)	8 (40)	20 (100)	**0.007**	0.054	** < 0.001**
		Short/hidden, n (%)	17 (85)	10 (50)	12 (60)	0 (0)			
	Restriction	Not restricted, n (%)	6 (30)	18 (90)	11 (55)	20 (100)	**0.001**	0.207	0.349
		Only base, n (%)	7 (35)	1 (5)	6 (30)	0 (0)			
		Restricted, n (%)	7 (35)	1 (5)	3 (15)	0 (0)			
	Calcification	Present, n (%)	3 (15)	5 (25)	11 (55)	7 (35)	0.429	**0.005**	0.490
		Absent, n (%)	17 (85)	15 (75)	9 (45)	13 (65)			
Anterior leaflet	Thickness	Not thickened, n (%)	4 (20)	5 (25)	4 (20)	10 (50)	0.705	1	0.102
		Thickened, n (%)	16 (80)	15 (75)	16 (80)	10 (10)			
	Restriction	Not restricted, n (%)	20 (100)	20 (100)	20 (100)	20 (100)	–	–	–
		Only base, n (%)	0 (0)	0 (0)	0 (0)	0 (0)			
		Restricted, n (%)	0 (0)	0 (0)	0 (0)	0 (0)			
	Calcification	Present, n (%)	2 (10)	5 (25)	4 (20)	3 (15)	0.212	0.376	0.429
		Absent, n (%)	18 (90)	15 (75)	16 (80)	17 (85)			
Annulus	AP–APm (absolute value)	< 3, n (%)	5 (25)	6 (30)	8 (40)	6 (30)	0.617	0.071	0.524
		≥ 3 and < 5, n (%)	4 (20)	6 (30)	8 (40)	9 (45)			
		≥ 5, n (%)	11 (55)	8 (40)	4 (20)	5 (25)			
	Calcification	Present, n (%)	6 (30)	3 (15)	9 (45)	5 (25)	0.256	0.327	0.429
		Absent, n (%)	14 (70)	17 (85)	11 (55)	15 (75)			
Chordae tendineae	Thickness	Not thickened, n (%)	4 (20)	12 (60)	5 (25)	8 (40)	**0.010**	0.705	0.206
		Thickened, n (%)	16 (80)	8 (40)	15 (75)	12 (60)			
Papillary muscles	Thickness	Not thickened, n (%)	5 (25)	11 (55)	12 (60)	15 (75)	0.053	0.025	0.185
		Thickened, n (%)	15 (75)	9 (45)	8 (40)	5 (25)			
Valve function	Stenosis	Absent/mild, n (%)	20 (100)	20 (100)	20 (100)	20 (100)	–	–	–
		Moderate/severe, n (%)	0 (0)	0 (0)	0 (0)	0 (0)			
	Regurgitation	Absent/mild, n (%)	5 (25)	6 (30)	9 (45)	11 (55)	0.742	0.120	0.488
		Moderate/severe, n (%)	15 (75)	13 (65)	11 (55)	9 (45)			
Aortic valve									
Cusps	Thickness	Not thickened, n (%)	3 (15)	7 (35)	4 (16)	8 (40)	0.144	0.677	0.744
		Thickened, n (%)	17 (85)	13 (65)	20 (80)	12 (60)			
	Restriction	Not restricted, n (%)	15 (75)	18 (90)	18 (90)	20 (100)	0.212	0.212	0.147
		Restricted, n (%)	5 (25)	2 (10)	2 (10)	0 (0)			
	Calcification	Present, n (%)	9 (45)	4 (20)	12 (60)	8 (40)	0.091	0.342	0.168
		Absent, n (%)	11 (55)	16 (80)	8 (40)	12 (60)			
Valve function	Stenosis	Absent/mild, n (%)	19 (95)	20 (100)	19 (95)	18 (90)	**0.002**	0.065	0.095
		Moderate/severe, n (%)	1 (5)	0 (0)	1 (5)	2 (10)			
	Regurgitation	Absent/mild, n (%)	8 (40)	8 (40)	7 (35)	15 (75)	0.598	0.944	0.041
		Moderate/severe, n (%)	12 (60)	1 (5)	13 (65)	5 (25)			
Tricuspid valve									
Septal leaflet	Thickness	Not thickened, n (%)	9 (45)	13 (65)	6 (30)	8 (40)	0.204	0.327	0.113
		Thickened, n (%)	11 (55)	7 (35)	14 (70)	12 (60)			
	Length	Normal, n (%)	16 (80)	18 (90)	17 (85)	19 (95)	0.517	0.597	0.548
		Short, n (%)	3 (15)	2 (10)	3 (15)	1 (5)			
		Hidden, n (%)	1 (5)	0 (0)	0 (0)	0 (0)			
	Restriction	Not restricted, n (%)	14 (70)	20 (100)	19 (95)	19 (95)	0.029	0.109	0.311
		Only base, n (%)	5 (25)	0 (0)	1 (5)	1 (5)			
		Restricted, n (%)	1 (5)	0 (0)	0 (0)	0 (0)			
	Calcification	Present, n (%)	1 (5)	0 (0)	0 (0)	2 (10)	0.311	0.311	0.147
		Absent, n (%)	19 (95)	20 (100)	20 (100)	18 (90)			
Antero-posterior leaflet	Thickness	Not thickened, n (%)	10 (50)	14 (70)	12 (60)	16 (80)	0.197	0.525	0.465
		Thickened, n (%)	10 (50)	6 (30)	8 (40)	4 (20)			
	Length	Normal, n (%)	17 (85)	19 (95)	19 (95)	20 (100)	0.292	0.292	0.311
		Short, n (%)	3 (15)	1 (5)	1 (5)	0 (0)			
		Hidden, n (%)	0 (0)	0 (0)	0 (0)	0 (0)			
	Restriction	Not restricted, n (%)	18 (90)	20 (100)	20 (100)	20 (100)	0.147	0.147	–
		Only base, n (%)	2 (10)	0 (0)	0 (0)	0 (0)			
		Restricted, n (%)	0 (0)	0 (0)	0 (0)	0 (0)			
	Calcification	Present, n (%)	2 (10)	0 (0)	0 (0)	0 (0)	0.147	0.147	–
		Absent, n (%)	18 (90)	20 (100)	20 (100)	20 (100)			
Annulus	Calcification	Present, n (%)	5 (25)	2 (10)	2 (10)	1 (5)	0.212	0.212	0.548
		Absent, n (%)	15 (75)	18 (90)	18 (90)	19 (95)			
Chordae tendineae	Thickness	Not thickened, n (%)	11 (55)	12 (60)	18 (90)	16 (80)	0.749	0.749	0.168
		Thickened, n (%)	9 (45)	8 (40)	2 (10)	4 (20)			
Papillary muscles	Thickness	Not thickened, n (%)	14 (70)	18 (90)	15 (75)	19 (95)	0.114	0.723	0.548
		Thickened, n (%)	6 (30)	2 (10)	5 (25)	1 (5)			
Valve function	Stenosis	Absent/mild, n (%)	20 (100)	20 (100)	20 (100)	20 (100)	–	–	–
		Moderate/severe, n (%)	0 (0)	0 (0)	0 (0)	0 (0)			
	Regurgitation	Absent/mild, n (%)	12 (60)	10 (50)	8 (40)	9 (45)	0.892	0.571	0.519
		Moderate/severe, n (%)	8 (40)	10 (50)	12 (60)	11 (55)			
Mitral valve score			8.0 (6.5–9.5)	6.3 (4.1–7.9)	6.0 (4.1–7.0)	5.0 (4.0–6.0)	**0.010**	**0.001**	0.081
Aortic valve score			1.5 (1.0–2.0)	1.0 (0–1.0)	2.0 (1.0–2.0)	1.0 (1.0–2.0)	0.030	0.904	0.165
Tricuspid valve score			5.5 (4.0–7.0)	4.0 (3.0–6.0)	6.0 (5.0–6.8)	4.0 (4.0–5.0)	0.157	0.883	1
Valve score			15.8 (13.6–17.4)	11.0 (9.3–14.9)	12.8 (11.1–14.4)	11.0 (9.1–13.0)	**0.004**	**0.009**	0.461

The Bonferroni correction was applied to account for multiple comparisons (n = 4); significant p values (< 0.017) are highlighted in bold

*AP* anteroposterior, *SAM* systolic anterior motion

### Correlates of valve score values

Among population characteristics listed in Table 2, no one displayed significant correlations with the valve score values in patients with ATTR–CA, and only with RA strain in ATTR–CA controls. Some significant correlations were found in patients with AL–CA, namely with cardiac biomarkers (NT-proBNP and hs-troponin T), LV systolic function (LV ejection fraction and GLS), as well as the LV mass-to-strain ratio, E/e′ ratio, tricuspid annular plane systolic excursion and systolic pulmonary artery pressure (Supplemental Table 2).

### The valve score to diagnose ATTR–CA

Valve score values had an AUC of 0.765 to discriminate between ATTR- and AL–CA, and the best cut-off was 14 [sensitivity 75%, specificity 70%, positive predictive value (PPV) 71%, negative predictive value (NPV) 74%]. Among all clinical, laboratory and echocardiographic characteristics, only age, interventricular septum and posterior wall thickness, LVMI, and continuous score values or values higher than or equal to 14 emerged as univariable predictors of ATTR–CA. None of these variables independently predicted ATTR–CA (Supplemental Table 3). Valve score values had an AUC of 0.739 to distinguish ATTR–CA from matched controls; the best cut-off was again 14, with the same sensitivity, specificity, PPV and NPV than for the differentiation between ATTR- and AL–CA. Absolute valve score values and the 14 cut-off were both univariable predictors, while they did not reach independent prognostic significance (Supplemental Table 4).

**Fig. 6:**
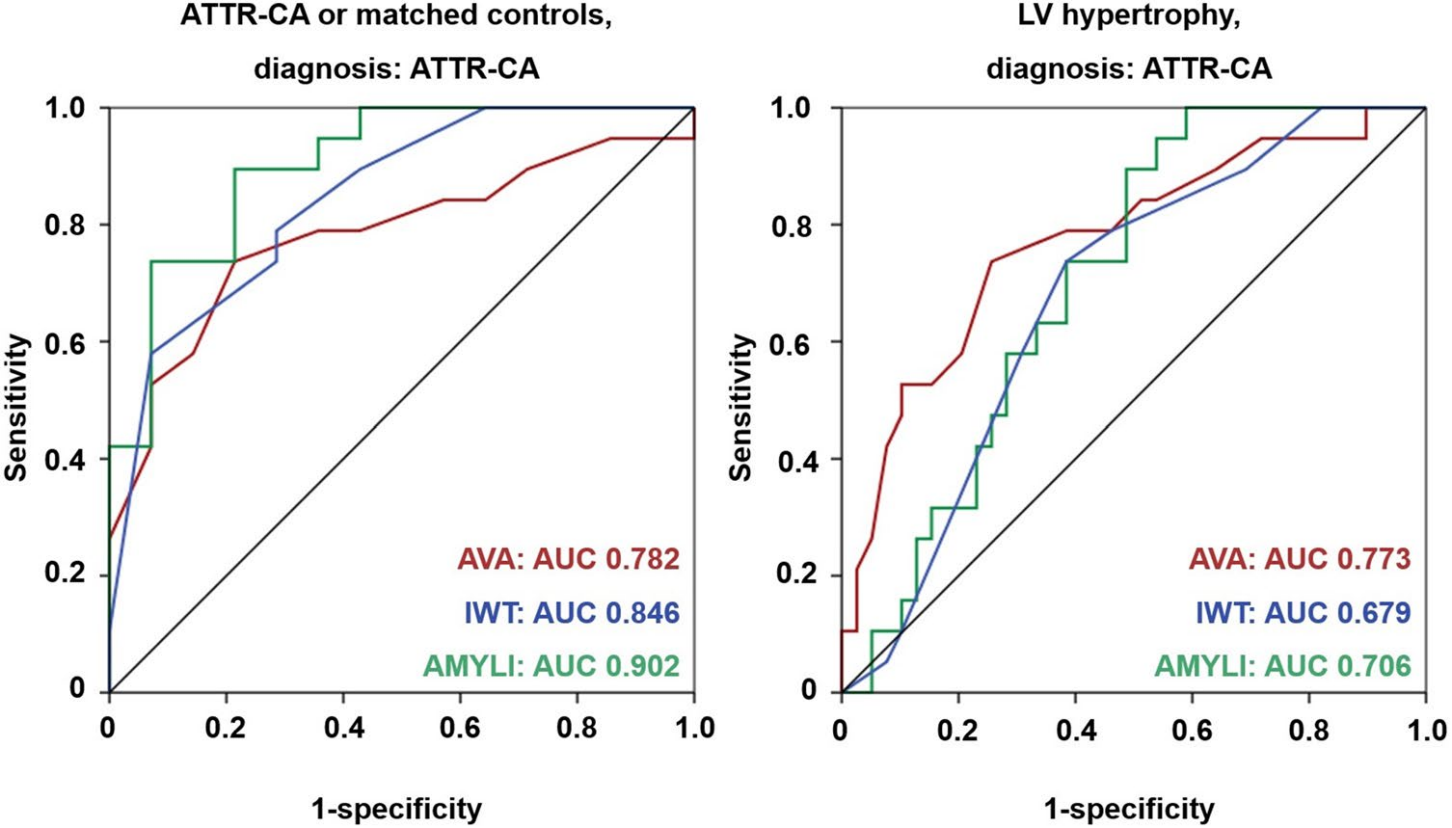
The valve, increased wall thickness (IWT), and AMYLI scores for the diagnosis of amyloid transthyretin cardiac amyloidosis (ATTR–CA). Area under the curve (AUC) values are reported. The scores were evaluated in patients with ATTR–CA or matched controls (*left*) or in those with left ventricular (LV) hypertrophy (*right*). p values for all comparisons were non-significant (ATTR–CA or controls: valve score vs. IWT, p = 0.524; valve score vs. AMYLI, p = 0.205; IWT vs. AMYLI, p = 0.313; LV hypertrophy: valve score vs. IWT, p = 0.243; valve score vs. AMYLI, p = 0.402; IWT vs. AMYLI, p = 0.704)

We also compared the valve score and 2 validated diagnostic scores (IWT and AMYLI). AUC values to diagnose ATTR–CA were 0.782, 0.846 and 0.902, respectively, in patients with ATTR–CA or matched controls, and 0.773, 0.679 and 0.706 in patients with LV hypertrophy (n = 67, 84%) (all non-significant p values) (Fig. 6).

### Outcome

Eleven patients with ATTR–CA (55%) died over 2.1 years (1.0–3.7); 6 patients out of 19 with available data (32%) died for cardiovascular causes. 10 Patients out of 18 (55%) were hospitalized for HF over 1.3 years (0.6–2.3). Among patients with AL–CA, 9 died (45%), 7 of whom for cardiovascular causes (35%), over 3.1 years (0.7–7.4); 9 patients out of 16 (56%) were hospitalized for HF over 0.4 years (0.2–1.2). The valve score was not a good predictor of fatal outcomes in ATTR–CA (AUC values 0.611 for all-cause death, 0.519 for cardiovascular death), while it was more predictive in AL–CA (AUC values 0.687 for all-cause death, 0.725 for cardiovascular death). AUC values for HF hospitalization were quite similar in ATTR–CA (0.700) and AL–CA (0.635).

## Discussion

The valve score is the first attempt to synthesize an in-depth description of multiple morphological and valve function parameters of CA patients in a score, easily derivable by both expert and novice echocardiographers. We report that patients with ATTR–CA have a greater impairment of MV structure and function, conditioning higher score values in this group. ATTR–CA patients more often displayed a shortened or hidden and retracted PMVL and a thickened mitral chordae tendineae compared with AL–CA patients, and at the same time patients with AL–CA displayed more often a short or hidden PMVL than their matched controls. While the score was designed with primarily descriptive purposes, we showed that it retains diagnostic and potentially prognostic potential.

### The valve score

The valve score was designed to allow a comprehensive evaluation of multiple valves, in agreement with the notion of CA as a systemic disorder. The more recognized feature of valvular involvement in CA is increased thickness of atrioventricular valve leaflets and sub-valvular apparatus. The thickness of each mitral and tricuspid leaflet and aortic cusps was explored. Restricted leaflet motion in the MV has been associated with valve dysfunction in CA, even if there is less consensus that it is a characteristic feature of CA compared to leaflets thickening [23]. In our analysis, this finding was confirmed, as the restriction of the posterior mitral leaflet was more frequent in patients with CA than in their matched controls. Restriction of the mitral and tricuspid leaflets was then also included in the score.

A particularly interesting aspect is the role of calcification. Even if the literature on this subject is scant, we assumed that a typical feature of CA should be thickening of the valvular leaflets without accompanying calcification, which usually represents an ominous sign in degenerative (broadly defined as “senile”) forms. Thus, we gave 1 point to the absence of calcification and 0 to its presence, as we deemed calcium deposits to be less determinant in causing CA-related valve dysfunction. Our results were in line with this assumption, as we report significantly less frequent calcification of the PMVL in ATTR–CA compared to their matched controls. Except for the tricuspid valve, calcification was less present in ATTR–CA cases compared to controls, although differences did not reach statistical significance possibly because of the small number of patients.

### Comparison between the valve score and other scores of valve disease

The valve score shares some similarities with other established echocardiographic score systems, especially with ones designed to assess the mitral valve. The most similar is the Wilkins score, which is still the most used in clinical practice [24]. This score provides an evaluation of calcification, thickness, and mobility of the anterior mitral leaflet and the thickness of the chordae tendineae. The valve score encompasses all these elements, but also includes an assessment of global valve function, the measure of the AP diameter of the mitral valve annulus, the presence of mitral annulus calcification and of papillary muscles thickness. At the same time, while each variable in the Wilkins score is scored from 1 to 4, our score involves a dichotomous assessment of these characteristics. This represents a necessity in order to maintain an acceptable degree of feasibility, considering that we included an assessment of not only the mitral valve, but of the aortic and tricuspid vale as well. At the same time, we thought that, considering most of our variables are qualitative assessment, a dichotomous choice would increase reproducibility. The subjectivity leading to interobserver variability is one of the main defects of Wilkins score, considering that all its variables can only be assessed semi-quantitatively. The Nunes score is similar to the valve score as both try to increase reproducibility by using only dichotomous variables, which include mitral valve area, the involvement of the subvalvular apparatus, the presence of anterior mitral leaflet displacement into the LV cavity and the commissural area ratio.

### The valve score to assess valvular involvement in cardiac amyloidosis

The greater valve involvement of ATTR–CA patients was mirrored by a significantly higher mean global valve score in this group (15.8 in patients with ATTR–CA compared with 11.0 in patients with AL–CA, p 0.004). This could be explained by the fact that ATTR–CA patients were older than the AL–CA group. However, we found no other significant clinical difference between our groups other than age, and even measures of global functional impairment, such as NYHA class, did not differ. The only notable difference was that patients with ATTR–CA had greater degrees of LV hypertrophy than their matched controls or even than patients with AL–CA. Another possible explanation is that the slower disease progression of ATTR–CA allows for the slow deposition of amyloid and thus the manifestation of valve dysfunction, a phenomenon that with AL–CA does not have time to realize. Lastly, it is possible that ATTR has a greater tropism for heart valves compared to light-chains, even if this hypothesis does not appear to be supported by autoptic histopathological studies assessing the amyloid burden in AL–CA heart valves [12, 25, 26].

ATTR–CA valve involvement was even more marked considering the mitral valve: indeed, ATTR–CA patients more often displayed a shortened or hidden and retracted PMVL and a thickened mitral chordae tendineae compared with AL–CA patients. In particular, considering only partial score results for the mitral valve, we found that ATTR–CA had higher mitral score values than those with AL–CA or than ATTR–CA controls. Additionally, patients with AL–CA displayed more often a short or hidden PMVL than their matched controls.

As for the aortic valve, we found a higher prevalence of valve stenosis in the ATTR–CA group than the AL–CA group, and a trend towards less frequent calcification in patients with ATTR–CA compared with those with AL–CA (p = 0.091). Differences between groups in the tricuspid valve scores did not reach statistical significance, perhaps reflecting a less severe involvement of the tricuspid valve in the disease process.

Beyond the description of individual valves, we reported significant differences also in terms of global score values: the total mean valve score was 15.8 in patients with ATTR–CA, 11.0 in those with AL–CA, 12.8 in ATTR–CA controls, and 11.0 in AL–CA controls (p values 0.004 for ATTR–CA vs. AL–CA, 0.009 for ATTR–CA vs. matched controls, and 0.461 for AL–CA vs. matched controls). The only instance where our score was not able to discriminate between groups was between AL–CA and matched controls. These results were driven by differences in mitral valve features.

### Diagnostic value of the valve score

Valve score values allowed to distinguish between ATTR- and AL–CA and between ATTR–CA and their matched control, with 14 as the best cut-off, and a fair diagnostic performance. The valve score, possibly integrated in a more comprehensive echocardiographic evaluation, may at least orient towards one of the two forms of CA, although it cannot replace other more specific tests such as the search for a monoclonal plasma component or diphosphonates scintigraphy. Interestingly, the performance of the valve score to diagnose ATTR–CA did not differ significantly from two echocardiographic diagnostic scores, i.e., the IWT and AMYLI scores.

### Prognostic implication

The valve score was not a good predictor of fatal outcomes in ATTR–CA, while it was slightly more predictive in AL–CA (AUC values 0.68 for all-cause death, 0.72 for cardiovascular death). AUC values for HF hospitalization were similar in ATTR–CA (0.70) and AL–CA (0.63). The prognostic value of the score is therefore quite limited but not entirely negligible, also considering the small number of patients considered and the lack of composite endpoints.

### Limitations

This hypothesis-generating study evaluated a small number of patients, none of whom with ATTRv.

We also focused on a single echocardiogram at the time of diagnosis, while follow-up echocardiograms might have provided valuable insights on valvular disease progression. The IWT and AMYLI scores were employed in a different way than originally proposed (i.e., to diagnose CA in patients with unexplained hypertrophy). Possible developments of this study are the identification of the diagnostic and prognostic yield of each score item, and the creation of two versions of the score (with a smaller number of items and unequal weighting) to be used for diagnosis or risk stratification.

Also, weighting of each item should be assessed in larger cohorts, as well as modified AP diameter.

## Conclusions

The valve score is a reproducible, easily derivable, assessment tool for valve disease in CA. Although valve morphology and function alone are not sufficient to diagnose CA or ATTR–CA, patients with ATTR–CA have a prominent impairment of mitral valve structure and function. Higher score values may then help identify patients with ATTR–CA among those with CA or with unexplained hypertrophy.

### Supplementary Information

Below is the link to the electronic supplementary material.

Supplementary file1 (DOCX 115 KB)

## Abbreviations

AL: Amyloid light-chain
AMYLI: AMYLoidosis Index
AP: Anteroposterior
ATTR: Amyloid transthyretin
AUC: Area under the curve
CA: Cardiac amyloidosis
CI: Confidence interval
CMR: Cardiovascular magnetic resonance
EMB: Endomyocardial biopsy
FTGM: Fondazione Toscana Gabriele Monasterio
GLS: Global longitudinal strain
HF: Heart failure
ICC: Intraclass correlation coefficient
IWT: Increased wall thickness
LV: Left ventricular
NPV: Negative predictive value
PPV: Positive predictive value
RWT: Relative wall thickness
