# Supplementary material for: Valve disease in cardiac amyloidosis: an echocardiographic score
Source: Int J Cardiovasc Imaging. 2023 Jun 21;39(10):1873–87. doi: 10.1007/s10554-023-02901-2 (PMC10589146; doi:10.1007/s10554-023-02901-2)
Supplement: Supplementary file 1 — Supplementary file1 (DOCX 115 KB) [file 10554_2023_2901_MOESM1_ESM.docx]

**Supplemental material**

**Methods: left atrial and right chamber strain**

LA strain analysis was performed on 4- and 2-chamber apical view grey-scale images^1^. We adopted the QRS method, whereby the LA endocardial border is manually traced at LV end-systole in both apical views. The software automatically generated a ROI including 6 segments with different colors per view. The ROI was manually adjusted to include the thickness of the LA myocardium and optimize tracking quality analysis. A curve was generated for each of the 12 atrial segments. Tracking quality was checked and manually corrected, when needed, to ensure optimal tracking; the pulmonary veins and LA appendage were not included. Only segments deemed appropriately and accurately tracked were considered for analysis. The peak LA longitudinal strain (LA-PALS), measured during ventricular systole (normal values 33.5±10.9%, lower reference limit 11.7 ^2^, was calculated as mean between 4- and 2-chamber measurements. RV myocardial deformation was assessed by 2D speckle-tracking imaging on the RV-focused apical 4-chamber view using the Tomtec Arena package. After manual tracing of the end-systolic RV endocardial border, a ROI was automatically generated; its width and position were manually adjusted to include the entire myocardial wall and to exclude the pericardium. Pulmonary valve closure was identified on the pulse-wave Doppler tracing of the RV outflow tract. The software automatically divides the RV free-wall and the interventricular septum. The quality of tracking was automatically validated by software and confirmed visually from the 2D images. Subjects in whom >2 segments per ventricle showed inadequate tracking despite attempts to readjust the ROI position and width were excluded from analysis^3^. Normal values of RV GLS are: −22.3±3.3% (lower reference limit -15.7%) in men and −20.7±2.9% (lower reference limit -14.9%) in women ^4^. The peak right atrial longitudinal strain (RA-PALS) was measured. Endocardial borders of the RA were traced on the end-diastolic and end-systolic frame in a focused 4-chamber apical view oriented to the right-sided chambers. End-diastole and end-systole were defined based on both electrocardiogram and visual assessment. All images were acquired with a frame rate of at least 50 frames/sec. For RA strain measurement, the zero reference was set at end-diastole. As for LA, RA-PALS was measured on 3 cardiac cycles and averaged were patients had atrial fibrillation, flutter or tachycardia.

**Laboratory evaluation**

As part of the diagnostic workup, and within 48 hours from the echocardiogram, blood samples were drawn in the morning after an overnight fasting period^11^. Plasma samples were stored at -20 °C and analyzed within 7 days. Estimated glomerular filtration rate was calculated through the Modification of Diet in Renal Disease (MDRD) equation^12^. NT-proBNP was measured through the electro-chemiluminescence binding assay (ECLIA) method on the Cobas e411 platform (Roche Diagnostics Italia, Monza, Italy)^13^. hs-TnT was measured through the Roche Diagnostics assay.

**Supplemental Figure 1. Valve score items: tricuspid valve.**


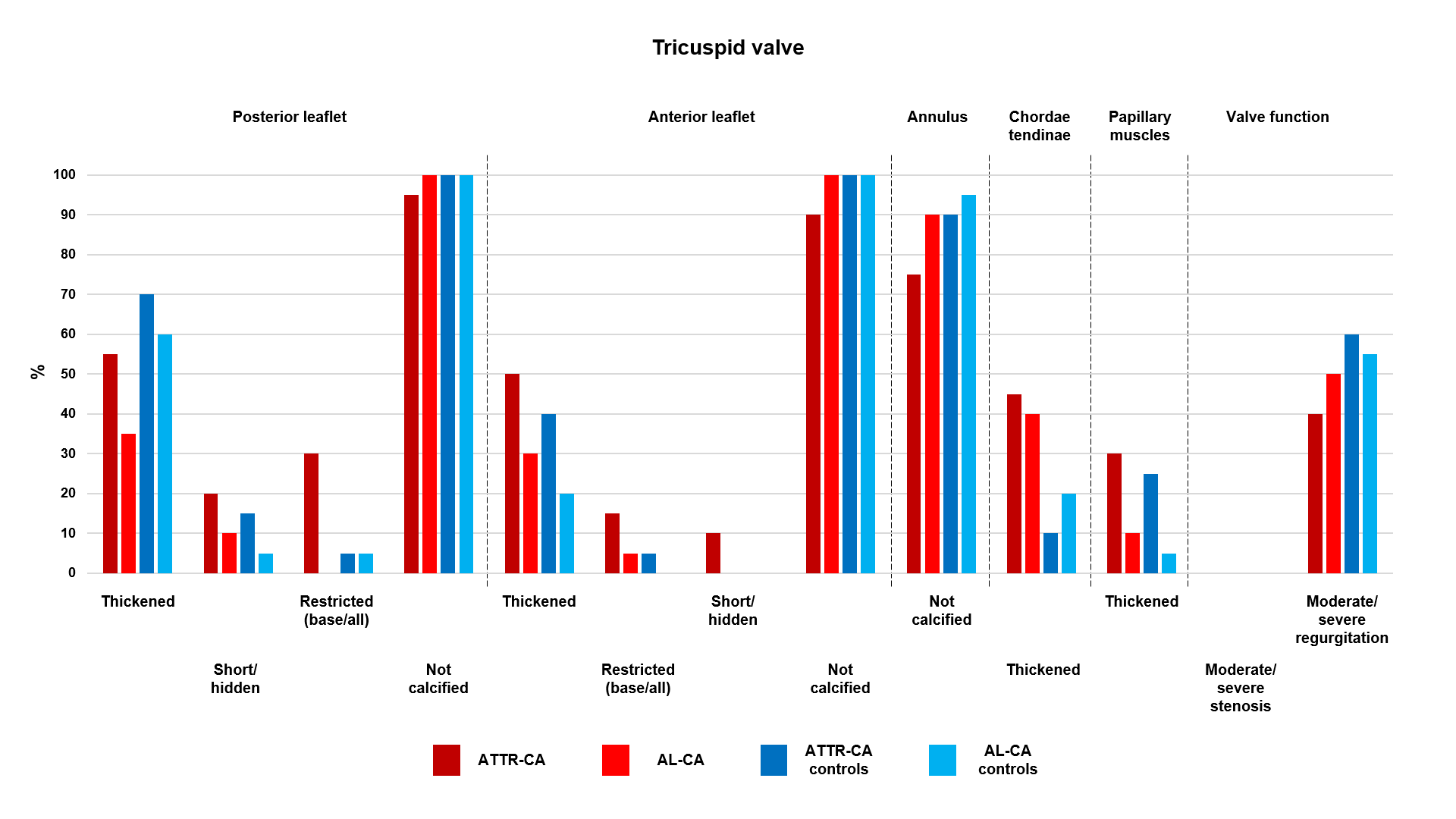


AL, amyloid light-chain; AP, antero-posterior; ATTR, amyloid transthyretin; CA, cardiac amyloidosis.

**Supplemental Table 1. The Increased Wall Thickness (IWT) score.**

|  | **Cut off** | **Points** |
| --- | --- | --- |
| **RWT** | > 0.6 | 3 |
| **E/e’** | >11 | 1 |
| **TAPSE**, *mm* | ≤ 19 | 2 |
| **GLS**, *%* | ≥ -13 | 1 |
| **SAB** | > 2.9 | 3 |

GLS, global longitudinal strain; RWT, relative wall thickness; SAB, systolic apex to base ratio; TAPSE, tricuspid annular plane systolic excursion.

**Supplemental Table 2. Correlates of AVA score values.**

|  | **ATTR-CA** | **AL-CA** | **ATTR-CA controls** | **AL-CA**  **controls** |
| --- | --- | --- | --- | --- |
| Age | p=0.233 | p=0.850 | p=0.782 | p=0.691 |
| Men | p=0.465 | p=0.510 | p=0.381 | p=0.567 |
| NYHA I/II/III/IV | p=0.065 | p=0.213 | p=0.119 | p=0.754 |
| Hypertension | p=0.639 | p=0.580 | p=0.484 | p=0.991 |
| Diabetes | p=0.763 | p=0.611 | p=0.186 | p=0.922 |
| NT-proBNP | p=0.380 | p=0.030  r=0.499 | p=0.044  r=0.486 | p=0.933 |
| hs-TnT | p=0.859 | p=0.043  r=0.497 | p=0.623 | p=0.273 |
| eGFR | p=0.847 | p=0.104 | p=0.181 | p=0.750 |
| Atrial fibrillation/flutter/tachycardia | p=0.874 | p=0.743 | p=0.256 | p=0.802 |
| IVS | p=0.359 | p=0.112 | p=0.964 | p=0.962 |
| PW | p=0.085 | p=0.092 | p=0.092 | p=0.915 |
| LVEDVi | p=0.774 | p=0.278 | p=0.459 | p=0.930 |
| LVESVi | p=0.999 | p=0.850 | p=0.539 | p=0.460 |
| LVEF | p=0.869 | p=0.017  r=0.528 | p=0.974 | p=0.314 |
| GLS | p=0.643 | p=0.024  r=0.544 | p=0.181 | p=0.421 |
| LVMI | p=0.174 | p=0.066 | p=0.658 | p=0.797 |
| MSR | p=0.842 | p=0.025  r=0.540 | p=0.449 | p=0.661 |
| RWT | p=0.179 | p=0.126 | p=0.984 | p=0.849 |
| E/e’ | p=0.651 | p=0.028  r=0.503 | p=0.155 | p=0.784 |
| LAVI | p=0.335 | p=0.113 | p=0.507 | p=0.510 |
| PALS | p=0.887 | p=0.126 | p=0.711 | p=0.689 |
| RV diameter | p=0.942 | p=0.586 | p=0.586 | p=0.816 |
| TAPSE | p=0.570 | p=0.032  r=0.493 | p=0.626 | p=0.774 |
| RV strain | p=0.632 | p=0.565 | p=0.953 | p=0.633 |
| Systolic PAP | p=0.524 | p=0.033  r=0.491 | p=0.630 | p=0.280 |
| RA diameter | p=0.793 | p=0.567 | p=0.601 | p=0.486 |
| RA strain | p=0.128 | p=0.816 | p=0.004  r=0.719 | p=0.373 |

AL, amyloid light-chain; ATTR, amyloid transthyretin; eGFR, estimated glomerular filtration rate; IVS, interventricular septum; LAVI, left atrial volume index; LVEF, left ventricular ejection fraction; LVEDVi, left ventricular end-diastolic volume index; LVESVi, left ventricular end-systolic volume index; LVMI, left ventricular mass index; NT-proBNP, N-terminal pro-B-type natriuretic peptide; NYHA, New York Heart Association; PAP, pulmonary artery pressure; PW, posterior wall; RA, right atrial; RV, right ventricular; RWT, relative wall thickness; TAPSE, tricuspid annular plane systolic excursion.

**Supplemental Table 3. Predictors of amyloid transthyretin cardiac amyloidosis (ATTR-CA) among patients with CA.**

|  | **Univariable analysis** | | **Multivariable analysis** | | | |
| --- | --- | --- | --- | --- | --- | --- |
|  | **p** | **OR (95% CI)** | **p** | **OR (95% CI)** | **p** | **OR (95% CI)** |
| Age | 0.030 | 1.13 (1.01-1.25) | 0.135 | - | 0.076 | - |
| Men | 0.226 | - | - | - | - | - |
| NYHA I/II/III/IV | 0.941 | - | - | - | - | - |
| Hypertension | 0.315 | - | - | - | - | - |
| Diabetes | 0.678 | - | - | - | - | - |
| NT-proBNP | 0.050 | - | - | - | - | - |
| hs-TnT | 0.471 | - | - | - | - | - |
| eGFR | 0.081 | - | - | - | - | - |
| Atrial fibrillation/flutter/tachycardia | 0.508 | - | - | - | - | - |
| IVS | 0.007 | 1.50 (1.12-2.01) |  | |  | |
| PW | 0.018 | 1.45 (1.07-1.98) |  |  |  |  |
| LVEDVi | 0.578 | - | - | - | - | - |
| LVESVi | 0.620 | - | - | - | - | - |
| LVEF | 0.822 | - | - | - | - | - |
| GLS | 0.479 | - | - | - | - | - |
| LVMI | 0.012 | 1.04 (1.01-1.06) | 0.127 | - | 0.178 | **-** |
| MSR | 0.386 | - | - | - | - | - |
| RWT | 0.112 | - | - | - | - | - |
| E/e’ | 0.729 | - | - | - | - | - |
| LAVI | 0.108 | - | - | - | - | - |
| PALS | 0.914 | - | - | - | - | - |
| RV diameter | 0.765 | - | - | - | - | - |
| TAPSE | 0.867 | - | - | - | - | - |
| RV strain | 0.182 | - | - | - | - | - |
| Systolic PAP | 0.668 | - | - | - | - | - |
| RA diameter | 0.734 | - | - | - | - | - |
| RA strain | 0.870 | - | - | - | - | - |
| AVA score | 0.007 | 1.36 (1.09-1.70) | 0.133 | - |  | |
| AVA score ≥14 | 0.006 | 7.00 (1.74-28.17) |  | | 0.116 | - |

AL, amyloid light-chain; ATTR, amyloid transthyretin; eGFR, estimated glomerular filtration rate; IVS, interventricular septum; LAVI, left atrial volume index; LVEF, left ventricular ejection fraction; LVEDVi, left ventricular end-diastolic volume index; LVESVi, left ventricular end-systolic volume index; LVMI, left ventricular mass index; NT-proBNP, N-terminal pro-B-type natriuretic peptide; NYHA, New York Heart Association; PAP, pulmonary artery pressure; PW, posterior wall; RA, right atrial; RV, right ventricular; RWT, relative wall thickness; TAPSE, tricuspid annular plane systolic excursion.

**Supplemental Table 4. Predictors of amyloid transthyretin cardiac amyloidosis (ATTR-CA) among patients with ATTR-CA or matched controls.**

|  | **Univariable analysis** | | **Multivariable analysis** | | | |
| --- | --- | --- | --- | --- | --- | --- |
|  | **p** | **OR (95% CI)** | **p** | **OR (95% CI)** | **p** | **OR (95% CI)** |
| Age | 1 | - | - | - | - | - |
| Men | 1 | - | - | - | - | - |
| NYHA I/II/III/IV | 0.584 | - | - | - | - | - |
| Hypertension | 0.433 | - | - | - | - | - |
| Diabetes | 1 | - | - | - | - | - |
| NT-proBNP | 0.293 | - | - | - | - | - |
| hs-TnT | 0.691 | - | - | - | - | - |
| eGFR | 0.616 | - | - | - | - | - |
| Atrial fibrillation/flutter/tachycardia | 0.744 | - | - | - | - | - |
| IVS | 0.003 | 1.94 (1.25-3.01) |  | |  | |
| PW | 0.002 | 4.17 (1.67-10.45) |  |  |  |  |
| LVEDVi | 0.135 | - | - | - | - | - |
| LVESVi | 0.861 | - | - | - | - | - |
| LVEF | 0.250 | - | - | - | - | - |
| GLS | 0.626 | - | - | - | - | - |
| LVMI | 0.011 | 1.03 (1.01-1.06) | 0.418 | - | 0.673 | **-** |
| MSR | 0.998 | - | - | - | - | - |
| RWT | 0.756 | - | - | - | - | - |
| E/e’ | 0.025 | 1.21 (1.02-1.42) | 0.739 | - | 0.867 | **-** |
| LAVI | 0.213 | - | - | - | - | - |
| PALS | 0.008 | 0.69 (0.53-0.91) | 0.036 | 0.74 (0.55-0.98) | 0.034 | 0.72 (0.53-0.98) |
| RV diameter | 0.767 | - | - | - | - | - |
| TAPSE | 0.689 | - | - | - | - | - |
| RV strain | 0.187 | - | - | - | - | - |
| Systolic PAP | 0.750 | - | - | - | - | - |
| RA diameter | 0.343 | - | - | - | - | - |
| RA strain | 0.076 | - | - | - | - | - |
| AVA score | 0.017 | 1.38 (1.06-1.80) | 0.757 | - |  | |
| AVA score ≥14 | 0.006 | 7.00 (1.74-28.17) |  | | 0.466 | - |

AL, amyloid light-chain; ATTR, amyloid transthyretin; eGFR, estimated glomerular filtration rate; IVS, interventricular septum; LAVI, left atrial volume index; LVEF, left ventricular ejection fraction; LVEDVi, left ventricular end-diastolic volume index; LVESVi, left ventricular end-systolic volume index; LVMI, left ventricular mass index; NT-proBNP, N-terminal pro-B-type natriuretic peptide; NYHA, New York Heart Association; PAP, pulmonary artery pressure; PW, posterior wall; RA, right atrial; RV, right ventricular; RWT, relative wall thickness; TAPSE, tricuspid annular plane systolic excursion.

**References (Supplemental material)**

1. Badano LP, Kolias TJ, Muraru D, Abraham TP, Aurigemma G, Edvardsen T, D'Hooge J, Donal E, Fraser AG, Marwick T, Mertens L, Popescu BA, Sengupta PP, Lancellotti P, Thomas JD and Voigt JU. Standardization of left atrial, right ventricular, and right atrial deformation imaging using two-dimensional speckle tracking echocardiography: a consensus document of the EACVI/ASE/Industry Task Force to standardize deformation imaging. *European heart journal Cardiovascular Imaging*. 2018;19:591-600.

2. Cameli M, Miglioranza MH, Magne J, Mandoli GE, Benfari G, Ancona R, Sibilio G, Reskovic Luksic V, Dejan D, Griseli L, Van De Heyning CM, Mortelmans P, Michalski B, Kupczynska K, Di Giannuario G, Devito F, Dulgheru R, Ilardi F, Salustri A, Abushahba G, Morrone D, Fabiani I, Penicka M, Katbeh A, Sammarco G, Esposito R, Santoro C, Pastore MC, Comenale Pinto S, Kalinin A, Pičkure Ž, Ažman Juvan K, Zupan Mežnar A, Coisne A, Coppin A, Opris MM, Nistor DO, Paakkanen R, Biering-Sørensen T, Olsen FJ, Lapinskas T, Vaškelyté JJ, Galian-Gay L, Casas G, Motoc AI, Papadopoulos CH, Loizos S, Ágoston G, Szabó I, Hristova K, Tsonev SN, Galli E, Vinereanu D, Mihaila Baldea S, Muraru D, Mondillo S, Donal E, Galderisi M, Cosyns B, Edvardsen T and Popescu BA. Multicentric Atrial Strain COmparison between Two Different Modalities: MASCOT HIT Study. *Diagnostics (Basel, Switzerland)*. 2020;10.

3. Muraru D, Onciul S, Peluso D, Soriani N, Cucchini U, Aruta P, Romeo G, Cavalli G, Iliceto S and Badano LP. Sex- and Method-Specific Reference Values for Right Ventricular Strain by 2-Dimensional Speckle-Tracking Echocardiography. *Circulation Cardiovascular imaging*. 2016;9:e003866.

4. Park JH. Two-dimensional Echocardiographic Assessment of Myocardial Strain: Important Echocardiographic Parameter Readily Useful in Clinical Field. *Korean circulation journal*. 2019;49:908-931.
